# Supplementary material for: Machine learning aided multiscale modelling of the HIV-1 infection in the presence of NRTI therapy
Source: PeerJ. 2023 Mar 31;11:e15033. doi: 10.7717/peerj.15033 (PMC10069423; doi:10.7717/peerj.15033)
Supplement: Supplemental Information 1 [file peerj-11-15033-s001.pdf]

Derivation of drug-specific parameters of equation (4)

According to Dixit and Perelson (2004), the single-dose version of equation (4) can be expressed as follows

$$C_b(t) = \frac{FDk_a}{V_d(k_e-k_a)}\left[e^{-k_a t} - e^{-k_e t}\right]. \quad \text{(S1)}$$

The well-known drug-specific parameters C_{max} (maximum concentration of a drug in the blood) AUC (area under the concentration-time curve) and t_{max} (when C_{max} occurs) can be derived from equation (S1) as

$$C_{max} = \frac{FD}{V_d}\left(\frac{k_e}{k_a}\right)^{\frac{k_e}{k_e-k_a}}, \quad \text{(S2)}$$

$$AUC = \int_0^{\infty} C_b(t)dt = \frac{FD}{V_d k_e}, \quad \text{(S3)}$$

$$t_{max} = \frac{ln\left(\frac{k_e}{k_a}\right)}{k_a-k_e}. \quad \text{(S4)}$$

C_{max}, AUC and t_{max} values are available for all NRTI drugs, and estimation of the pharmacokinetic parameters k_a and k_e can be done by solving equations (S2)-(S4) in the least-squares sense. In this way, we have evaluated k_a and k_e values of all drugs with the use of experimental C_{max}, AUC and t_{max} presented in Supplementary Table S1 with their references.
