# Supplementary material for: Machine learning aided multiscale modelling of the HIV-1 infection in the presence of NRTI therapy
Source: PeerJ. 2023 Mar 31;11:e15033. doi: 10.7717/peerj.15033 (PMC10069423; doi:10.7717/peerj.15033)
Supplement: Supplemental Information 2 — Mean square error (MSE) and linear correlation coefficient (R) for artificial neural network (ANN) models with different topologies (number of nodes per layer) for predicting logarithmic fold-change of IC50 values for the six NRTIs. Genotype-phenotype data of each NRTI is split as external test set and internal train-validation-test sets with 15% and 85%. Internal data sets are divided into training, validation, and test sets (80%, 10%, and %10). Internal data set is used for training and model selection. In the model selection process, we trained 20 models four times, and every 20 models have been tested on the internal test sets, and the models giving the best MSE scores are selected for each NRTI. Thus, four models are averaged to get the final model for each inhibitor. The selected models are tested on the external test set, and the resulting performance metrics are presented below. According to the table, two topologies yield relatively better results: the model has one layer with five neurons, and the model has two layers and three neurons in each layer. To make the model under consideration simple enough, we chose the model with a five-neuron hidden layer. [file peerj-11-15033-s002.docx]

**Table S1.** Mean square error (MSE) and linear correlation coefficient (R) for artificial neural network (ANN) models with different topologies (number of nodes per layer) for predicting logarithmic fold-change of ${IC}_{50}$ values for the six NRTIs. Genotype-phenotype data of each NRTI is split as external test set and internal train-validation-test sets with 15% and 85%. Internal data sets are divided into training, validation, and test sets (80%, 10%, and %10). Internal data set is used for training and model selection. In the model selection process, we trained 20 models four times, and every 20 models have been tested on the internal test sets, and the models giving the best MSE scores are selected for each NRTI. Thus, four models are averaged to get the final model for each inhibitor. The selected models are tested on the external test set, and the resulting performance metrics are presented below. According to the table, two topologies yield relatively better results: the model has one layer with five neurons, and the model has two layers and three neurons in each layer. To make the model under consideration simple enough, we chose the model with a five-neuron hidden layer.

| ANN Topology/Drug | 3TC | | ABC | | AZT | | D4T | | | DDI | | TDF | | Mean | |
| --- | --- | --- | --- | --- | --- | --- | --- | --- | --- | --- | --- | --- | --- | --- | --- |
|  | MSE | R | MSE | R | MSE | R | MSE | | R | MSE | R | MSE | R | MSE | R |
| 2 | 0.11 | 0.93 | 0.05 | 0.87 | 0.18 | 0.89 | | 0.02 | 0.92 | 0.02 | 0.90 | 0.05 | 0.73 | 0.070 | 0.872 |
| 3 | 0.09 | 0.94 | 0.04 | 0.88 | 0.19 | 0.88 | | 0.02 | 0.93 | 0.02 | 0.89 | 0.05 | 0.73 | 0.067 | 0.875 |
| 4 | 0.08 | 0.95 | 0.05 | 0.87 | 0.19 | 0.88 | | 0.02 | 0.92 | 0.02 | 0.89 | 0.05 | 0.73 | 0.068 | 0.872 |
| 5 | 0.08 | 0.95 | 0.04 | 0.88 | 0.19 | 0.88 | | 0.02 | 0.92 | 0.02 | 0.89 | 0.05 | 0.73 | **0.067** | **0.876** |
| 6 | 0.08 | 0.95 | 0.04 | 0.89 | 0.19 | 0.88 | | 0.02 | 0.92 | 0.02 | 0.87 | 0.05 | 0.73 | 0.067 | 0.874 |
| 7 | 0.08 | 0.95 | 0.04 | 0.89 | 0.19 | 0.88 | | 0.02 | 0.92 | 0.02 | 0.87 | 0.05 | 0.73 | 0.068 | 0.872 |
| $2\times2$ | 0.08 | 0.95 | 0.05 | 0.87 | 0.19 | 0.88 | | 0.02 | 0.92 | 0.02 | 0.87 | 0.05 | 0.73 | 0.069 | 0.869 |
| $3\times3$ | 0.08 | 0.95 | 0.05 | 0.87 | 0.19 | 0.88 | | 0.02 | 0.91 | 0.02 | 0.87 | 0.04 | 0.81 | **0.067** | **0.881** |
| $4\times4$ | 0.08 | 0.95 | 0.05 | 0.86 | 0.19 | 0.88 | | 0.02 | 0.90 | 0.02 | 0.87 | 0.04 | 0.80 | 0.069 | 0.875 |
| $5\times5$ | 0.08 | 0.95 | 0.05 | 0.86 | 0.19 | 0.88 | | 0.02 | 0.90 | 0.02 | 0.87 | 0.04 | 0.80 | 0.069 | 0.875 |
| $3\times3\times3$ | 0.08 | 0.95 | 0.05 | 0.86 | 0.19 | 0.88 | | 0.02 | 0.90 | 0.02 | 0.87 | 0.04 | 0.80 | 0.069 | 0.875 |
| $5\times5\times5$ | 0.08 | 0.95 | 0.05 | 0.86 | 0.19 | 0.88 | | 0.02 | 0.91 | 0.02 | 0.86 | 0.04 | 0.80 | 0.069 | 0.875 |
