# Supplementary material for: Machine learning aided multiscale modelling of the HIV-1 infection in the presence of NRTI therapy
Source: PeerJ. 2023 Mar 31;11:e15033. doi: 10.7717/peerj.15033 (PMC10069423; doi:10.7717/peerj.15033)
Supplement: Supplemental Information 3 — Mean square error (MSE), linear correlation coefficient (R) and area under the curve (AUC) metric values for linear regression (LR) and artificial neural network (ANN) models for predicting fold-change values in IC50 values of the six NRTIs. [file peerj-11-15033-s003.docx]

**Table S1.** Mean square error (MSE), linear correlation coefficient (R) and area under the curve (AUC) metric values for linear regression (LR) and artificial neural network (ANN) models for predicting fold-change values in ${IC}_{50}$ values of the six NRTIs.

| Metric/Drug | 3TC | | ABC | | AZT | | D4T | | DDI | | TDF | | Mean | |
| --- | --- | --- | --- | --- | --- | --- | --- | --- | --- | --- | --- | --- | --- | --- |
|  | LR | ANN | LR | ANN | LR | ANN | LR | ANN | LR | ANN | LR | ANN | LR | ANN |
| MSE | 2.02 | **0.06** | 0.38 | **0.02** | 1.11 | **0.12** | 0.14 | **0.01** | 0.09 | **0.01** | 0.11 | **0.03** | 0.64 | **0.04** |
| R | 0.93 | **0.95** | 0.92 | 0.92 | 0.88 | **0.91** | 0.90 | **0.92** | 0.88 | 0.88 | 0.78 | **0.84** | 0.88 | **0.90** |
| AUC | 0.97 | **0.98** | **0.97** | 0.96 | 0.97 | **0.98** | 0.96 | **0.97** | **0.96** | 0.92 | 0.93 | **0.96** | 0.96 | 0.96 |
