# Supplementary material for: Machine learning aided multiscale modelling of the HIV-1 infection in the presence of NRTI therapy
Source: PeerJ. 2023 Mar 31;11:e15033. doi: 10.7717/peerj.15033 (PMC10069423; doi:10.7717/peerj.15033)
Supplement: Supplemental Information 4 [file peerj-11-15033-s004.docx]

**Table S2.** Pharmacokinetic parameters of the nucleotide reverse transcriptase inhibitors.

| Parameter/Drug | 3TC | ABC | AZT | D4T | DDI | TDF |
| --- | --- | --- | --- | --- | --- | --- |
| ${IC}_{50}$  Range  ($\times{10}^{-5}$ mg/ml) | [0.04-343.89] | [105.94-166.07] | [0.26-13.09] | [0.20-89.68] | [56.55-226.23] | [1.14-229.77] |
| ${IC}_{50}$  Geometric Mean  ($\times{10}^{-5}$ mg/ml) | 3.97 | 132.64 | 1.87 | 4.25 | 113.11 | 16.24 |
| $D$ (mg) | 300 | 300 | 300 | 40 | 400 | 300 |
| $I_{d}$ (day) | 1 | 0.5 | 0.5 | 0.5 | 1 | 1 |
| $F$ | 0.86 | 0.83 | 0.64 | 0.86 | 0.42 | 0.39 |
| $k_{a}$ (Estimated) | 27.98 | 51.07 | 37.42 | 54.29 | 32.34 | 8.36 |
| $k_{e}$ (Estimated) | 3.44 | 8.52 | 14.25 | 7.84 | 47.30 | 16.58 |
| $V_{d}$ (ml) | 91000 | 60200 | 112000 | 46000 | 54000 | 87500 |
| Considerable Side-Effects^*^ | Neutropenia (rare) | Hypersensitivity reaction, which may  be characterized by fever, rash,  myalgias, arthralgias, malaise | Nausea, headache, rash, anemia,  leukopenia, elevation of liver enzyme  levels, elevation of lactic acid level,  elevation of CPK level | Reversible peripheral neuropathy,  lactic acid elevation (rarely fatal) | GI intolerance, pancreatitis, gout,  reversible peripheral neuropathy | GI upset, low phosphate  levels |
| Reference | https://www.accessdata.fda.gov/ | https://www.accessdata.fda.gov/ | https://www.accessdata.fda.gov/ | https://www.accessdata.fda.gov/ | https://www.hiv-druginteractions.org/ | https://www.accessdata.fda.gov/ |

- Side-effects are taken from Table 1 of the paper “Montessori V, Press N, Harris M, Akagi L, Montaner JS. 2004. Adverse effects of antiretroviral therapy for HIV infection. CMAJ 170:229–238”.
