# Supplementary figures and images for: Machine learning aided multiscale modelling of the HIV-1 infection in the presence of NRTI therapy
Source: PeerJ. 2023 Mar 31;11:e15033. doi: 10.7717/peerj.15033 (PMC10069423; doi:10.7717/peerj.15033)

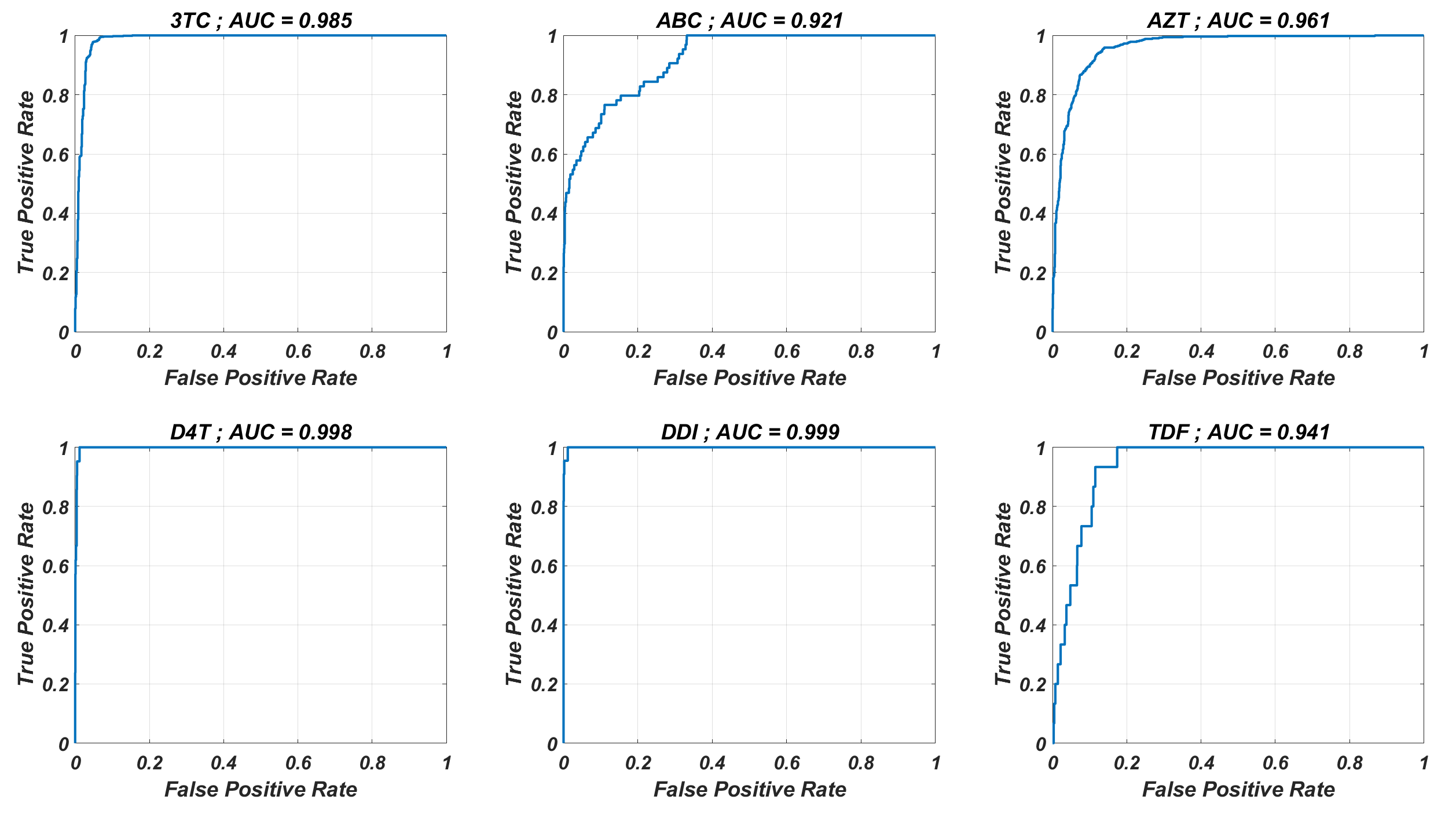

Supplement: Supplemental Information 5 — The ANN models are used to classify the given strains as resistant (Fold Change ≥ 3) and susceptible (Fold Change<3). The corresponding receiver operating characteristic curves with area under the curve (AUC) values are presented for each NRTI. [file peerj-11-15033-s005.png]

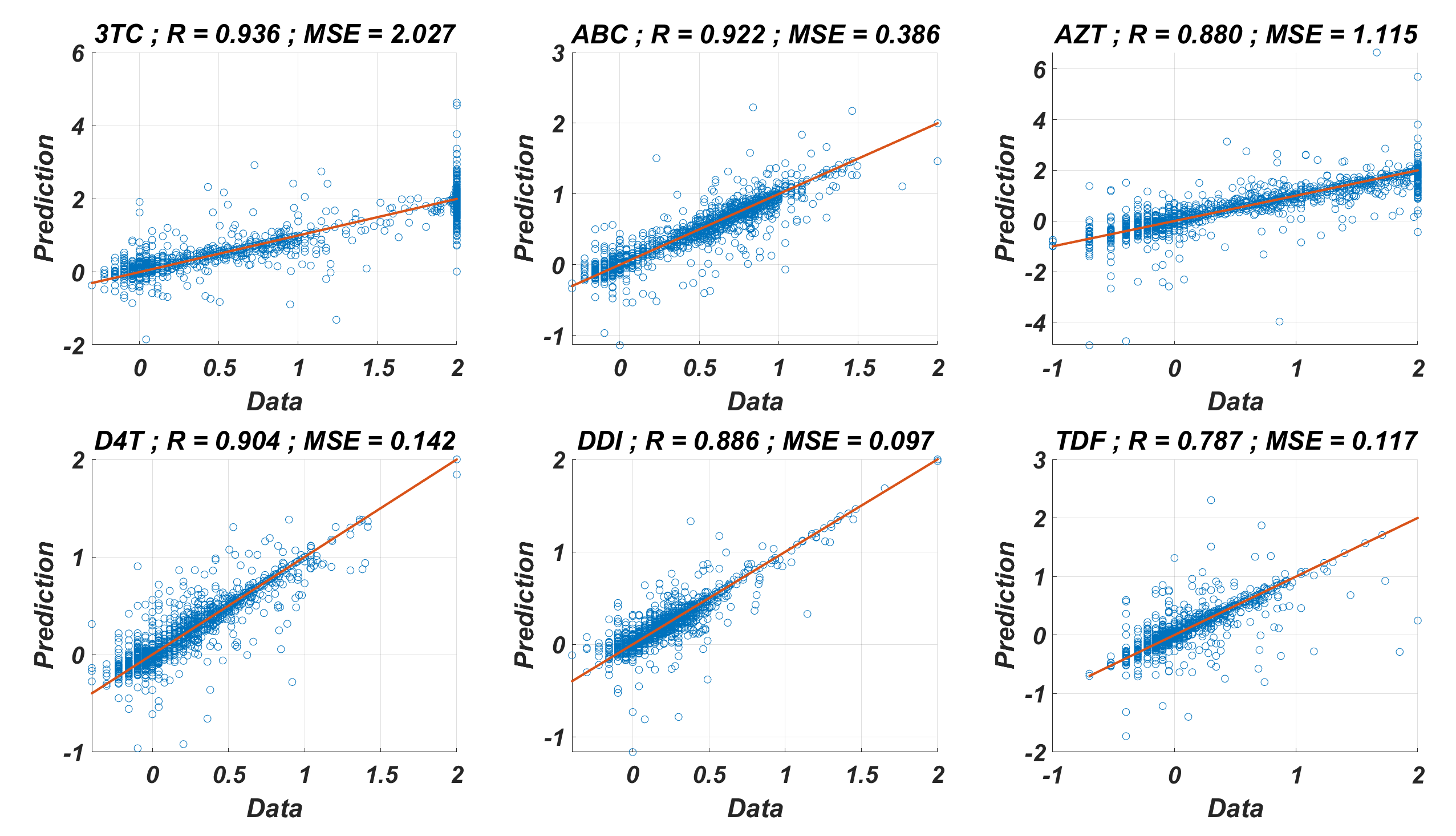

Supplement: Supplemental Information 6 — The x-axis of the figures denotes logarithmic fold change value for all existing mutant strains in the data and y-axis denotes corresponding predictions of the MLR models. For each MLR model, linear correlation coefficient (R) and mean square error (MSE) metrics are specified to measure the ability of these models to fit the existing real data. [file peerj-11-15033-s006.png]

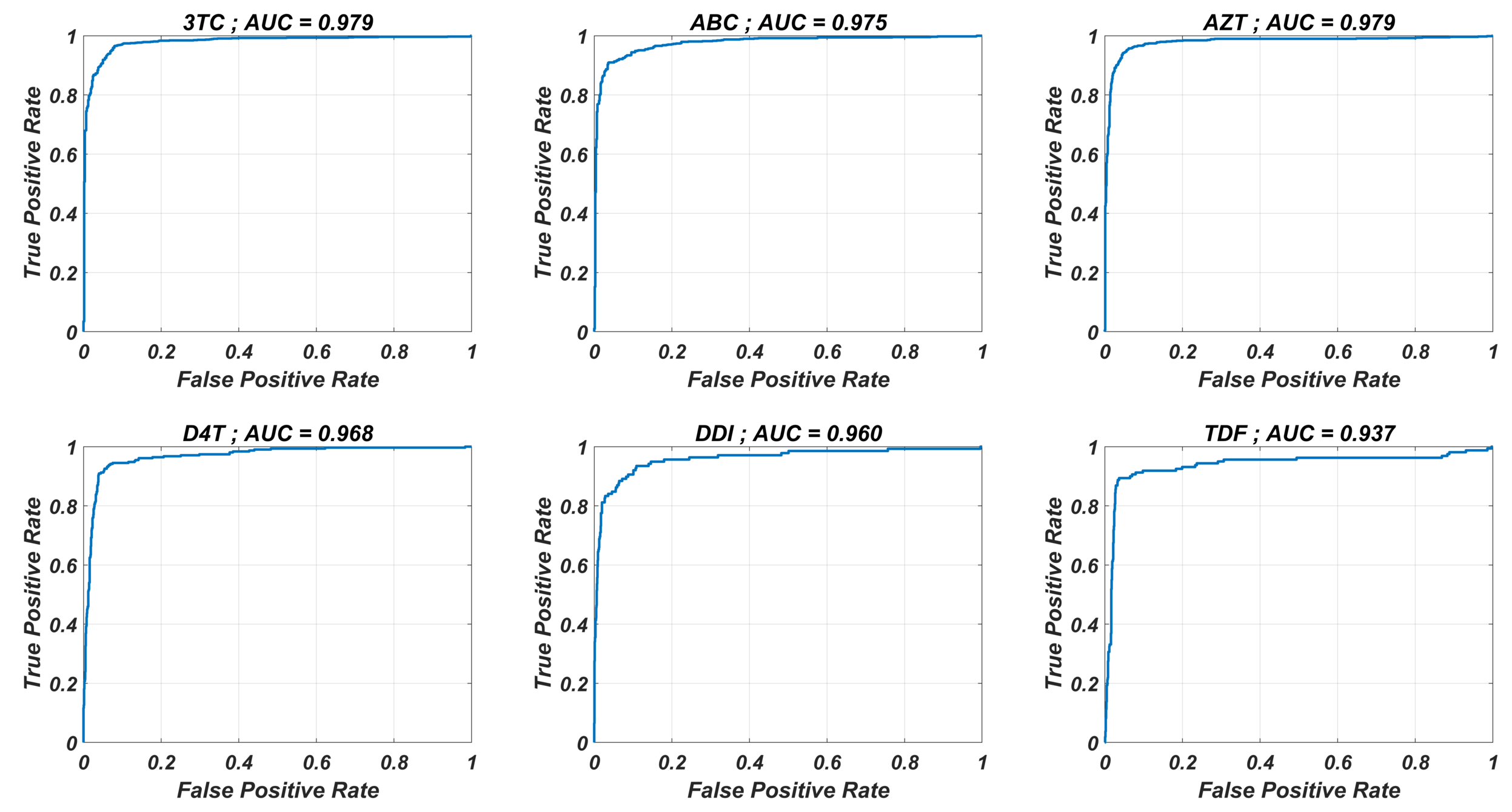

Supplement: Supplemental Information 7 — The MLR models are used to classify the given strains as resistant (Fold Change ≥ 3) and susceptible (Fold Change<3). The corresponding receiver operating characteristic curves with area under the curve (AUC) values are presented for each NRTI. [file peerj-11-15033-s007.png]
